# Bioimage analysis workflows: community resources to navigate through a complex ecosystem

**DOI:** 10.12688/f1000research.52569.1

**Published:** 2021-04-26

**Authors:** Perrine Paul-Gilloteaux, Sébastien Tosi, Jean-Karim Hériché, Alban Gaignard, Hervé Ménager, Raphaël Marée, Volker Baecker, Anna Klemm, Matúš Kalaš, Chong Zhang, Kota Miura, Julien Colombelli

**Affiliations:** 1 Université de Nantes, CNRS, INSERM, l’institut du thorax, Nantes, F-44000, France; 2 Université de Nantes, CHU Nantes, Inserm, CNRS, SFR Santé, Inserm UMS 016, CNRS UMS 3556, Nantes, F-44000, France; 3 Institute for Research in Biomedicine, IRB Barcelona, Barcelona Institute of Science and Technology, BIST, Barcelona, Spain; 4 Cell Biology and Biophysics Unit, European Molecular Biology Laboratory, Heidelberg, 69117, Germany; 5 Hub de Bioinformatique et Biostatistique, Département Biologie Computationnelle, Institut Pasteur, USR 3756, CNRS, Paris, 75015, France; 6 CNRS, UMS 3601, Institut Français de Bioinformatique, IFB-core, Evry, 91000, France; 7 Montefiore Institute, University of Liège, Liège, Belgium; 8 Montpellier Ressources Imagerie, BioCampus Montpellier, CNRS, INSERM, University of Montpellier, Montpellier, F-34000, France; 9 BioImage Informatics Facility, SciLifeLab, Stockholm, Sweden; 10 Computational Biology Unit, Department of Informatics, University of Bergen, Bergen, Norway; 11 Department of Information and Communication Technologies, University Pompeu Fabra, Barcelona, Spain; 12 Nikon Imaging Center, University of Heidelberg, Heidelberg, Germany

**Keywords:** Bioimage analysis, workflows, components, collections, FAIR principles, NEUBIAS, remote computing, scientific workflow management systems, knowledge database

## Abstract

Workflows are the keystone of bioimage analysis, and the NEUBIAS (Network of European BioImage AnalystS) community is trying to gather the actors of this field and organize the information around them.  One of its most recent outputs is the opening of the F1000Research NEUBIAS gateway, whose main objective is to offer a channel of publication for bioimage analysis workflows and associated resources. In this paper we want to express some personal opinions and recommendations related to finding, handling and developing bioimage analysis workflows.

The emergence of "big data” in bioimaging and resource-intensive analysis algorithms make local data storage and computing solutions a limiting factor. At the same time, the need for data sharing with collaborators and a general shift towards remote work, have created new challenges and avenues for the execution and sharing of bioimage analysis workflows.

These challenges are to reproducibly run workflows in remote environments, in particular when their components come from different software packages, but also to document them and link their parameters and results by following the FAIR principles (Findable, Accessible, Interoperable, Reusable) to foster open and reproducible science.

In this opinion paper, we focus on giving some directions to the reader to tackle these challenges and navigate through this complex ecosystem, in order to find and use workflows, and to compare workflows addressing the same problem. We also discuss tools to run workflows in the cloud and on High Performance Computing resources, and suggest ways to make these workflows FAIR.

## Introduction

Workflows are the keystone of bioimage analysis, ^1^^,^^2^ and the NEUBIAS community is trying to gather the actors of this field and organize the information around them. One of its most recent outputs is the opening of the F1000Research NEUBIAS gateway, whose main objective is to offer a channel of publication for bioimage analysis workflows ^3^ and associated resources.

In this paper, we aim to express some personal opinions and recommendations related to finding, handling and developing bioimage analysis workflows.

A bioimage analysis workflow is defined as a set of computational tools assembled in a specific order to process bioimages and estimate some parameters relevant to the biological system under study. To classify these computational tools, in the NEUBIAS community, we have defined these terms: *workflows*, *components* and *collections* ^1^^,^^4^ as follows. A *workflow* is built as a sequence of *components* coming from one or multiple software packages. It takes an image as input and outputs processed images, numerical values and/or annotations (e.g. biological objects outlines). A *component* is the software implementation of an image processing or analysis algorithm. We call *collection* the software package that gathers *components* and can be in the form of a generalist software platform such as ImageJ and Fiji, ^5^ Icy, ^6^ CellProfiler: ^7^ specialized platforms, such as analyzing a specific modality of microscopy e.g. super resolution image data; ^8^ or computationally optimized libraries such as ImgLib2 ^9^ or ITK. ^10^ Most of the time, standalone components cannot solve complex bioimage analysis problems on their own – that is why they need to be carefully assembled.

The emergence of resource-intensive analysis algorithms, e.g. supervised machine learning with convolutional neural networks, and of "big data” in bioimaging make local data storage and computing solutions a limiting factor. At the same time, the need for data sharing with collaborators and a general shift towards remote work, have created new challenges and avenues for the execution and sharing of bioimage analysis workflows.

These challenges are to reproducibly run workflows in remote environments, in particular when their components come from different *collections*, but also to document them and link their parameters and results by following the FAIR principles (Findable, Accessible, Interoperable, Reusable) ^11^ to foster open and reproducible science.

In this opinion paper we focus on giving some directions to the reader to tackle these challenges and navigate through this complex ecosystem, in order to find and use workflows (and *components*), and to compare workflows addressing the same problem. We also discuss tools to run workflows in the cloud and on High Performance Computing (HPC) resources, and suggest ways to make these workflows FAIR.

## Finding workflows or components for a specific biological problem or task

The first challenge in the creation of a workflow is to avoid duplicating the effort and being able to easily find and customize a workflow that has been used for a similar biological problem. Today, browsing the documentation of bioimage analysis tools, or asking a specific question in a generic forum, such as the newly created Image.sc forum, ^12^ will help guide the biologist or microscopist to existing tools. We believe that while this can be a good starting point it may not be sufficient. The NEUBIAS training courses ^13^^,^^14^ and the NEUBIAS Academy (see ^15^ in this Gateway) are two of the educational resources that can also help finding and adapting existing workflows. Exposing tools and workflows in a knowledge database has also been identified as very useful by the community. Table 1 illustrates some examples of such databases where bioimage analysts can reference their workflows using the proposed standardized framework and vocabulary in order to make them findable.

**Table 1.** Workflow finder. Some examples of such databases where bioimage analysts can reference their workflows.

Workflow finder	Target audience	Link
BIII	Bio Image Analyst, Biologist, Software developer	https://biii.eu
bio.tools	Bioinformatics/Computational Biology	https://bio.tools
Quantitative-plant	Plant Biologist	https://www.quantitative-plant.org/
Bio Image Model Zoo	Bio Image Analyst, Biologist, Focused on AI pretrained models	https://bioimage.io

BIII, the BioImage Informatics Index, has been created in the context of the NEUBIAS network and with the effort of tens of volunteers. Software tools (>1343), image databases for benchmarking (>24) and training materials (>71) for bioimage analysis are referenced and curated following standards constructed by the community. The range of software tools available includes workflows (>172), specific components (>898), and collections (>302). All entries are exposed following FAIR principles and accessible for other usage. They are described using Edam Bio Imaging, ^16^ a dedicated extension of the generalist EDAM ontology ^17^ for bioimage analysis, bioimage informatics, and bioimaging. It is developed in a community spirit, in collaboration between numerous bioimaging experts and ontology developers. It is used in BIII to describe the applications of these tools, by describing the operations performed (such as segmentation, visualization, or lower level operation) and the field of applications of these tools such as the imaging modalities to which it can be applied (i.e. EDAM Bioimaging Ontology). EDAM Bioimaging has now a solid basis. This basis is incrementally defined at specific meetings (i.e. taggathons) where suggestions for new terms, crowd-sourced from free tags by BIII users, are inspected and moderated for inclusion, or contrasted by bioimage analysis experts when no term is found adequate.

Similar initiatives exist, either for a broader range of applications, for example bio.tools, ^18^^,^^19^ which has gathered more than 20000 software tools in the full range of life science applications, or for more specific application topics, for example Quantitative Plant, which focusses on tools for the analysis of image data of plants ^20^ or BioImage.io for pre trained AI deep learning models.

By feeding the description of a workflow in the knowledge database BIII (following the recommendation provided), and thanks to workflow/tools interoperability standards, these workflows can be found by other bioimage analysts or automatically discovered and consumed by other registries, such as bio.tools, to reach a broader community.

## Comparing workflows

Once a candidate workflow has been found, the natural question is then if it is the best solution for the particular task one wants to solve. Table 2 shows three examples of resources comparing workflows.

**Table 2.** Example of websites to compare existing workflows on reference datasets

Benchmarking site	Link	Purpose
BIAFLOWS	https://biaflows.neubias.org/#/projects	Allows live testing of workflows
Grand-Challenges	https://grand-challenge.org/challenges/	Lists open challenge and results
Kaggle	https://www.kaggle.com/c/data-science-bowl-2018	One shot challenge for nuclei. Very generalist challenge platform

BIAFLOWS ^21^ is an open-source web platform to reproducibly deploy and publicly benchmark image analysis workflows with a strong focus on microscopy bioimages. The database stores scientific datasets, metadata, and versioned image analysis workflows with parameters optimized for the corresponding datasets. The workflows can be run remotely. The results (e.g. object annotations) from different workflows (or from runs with different parameter values) can be visualized remotely as an overlay on the original images. When the images hold reference annotations, the results are automatically benchmarked by commonly adopted benchmark metrics targeting one of the nine currently supported problem classes. The benchmark metrics of each workflow run can be browsed per image or as overall statistics over whole datasets. BIAFLOWS brings an automated mechanism leveraging DockerHub to encapsulate, version and make the workflows and their complete execution environment available upon every new release. Overall, BIAFLOWS enables integration and web-based evaluation of heterogeneous workflows originally written for diverse languages and libraries.

The Grand Challenge is a website cataloguing a set of challenges, focusing mostly on medical imaging. These challenges are usually hosted by a conference such as IEEE ISBI and run as an annual edition with specific reporting ^22^^,^^23^ and they gather and evaluate competing workflows to solve a common bioimage analysis task. In the microscopy imaging communities, a particular effort has gone towards nuclei segmentation with the goal of developing a universal nuclei segmenter that works across different imaging modalities, as for instance with the Kaggle Data Science Bowl of 2018, providing a considerable amount of annotated data. ^24^

## Towards reproducibility and interoperability in bioimage analysis

The current paradigm for bioimage analysts is to create workflows using a single platform or application, aka *collection*, for example Fiji, ^25^ CellProfiler, ^7^ or Icy. ^6^

By allowing the possibility to script a workflow calling their components with simplified programming language, these platforms offer ways to share and document the workflows for other users. Besides script creation, there are also options to create sharable elements with no programming skills, as detailed in. ^26^ This only requires the deployment of the software package to be run.

This reliance on graphical user interfaces favors the development of components built for a single *collection.* While this has stimulated the gathering of active communities around these *collections*, the coexistence of many multifunctional collections that are developed independently is not ideal for cloud deployment and FAIR principles. The graphical user interfaces are often not compatible with the type of remote computing offered by cloud technologies and the large collections contain largely overlapping components that are however not interoperable with each other. These *collections* therefore do not offer a unified and granular way of describing an image processing workflow. This situation also often requires users to learn multiple platforms to be able to complete their workflows. Code notebooks such as CodeOcean capsules or Jupyter notebooks offers also an easy access to cloud computing or HPC, but several aspect of workflow management are also left to the user, in particular data provenance.

At the same time that the field is shifting to running workflows in the cloud or high performance computing environments, there also comes the need to run more complex workflows integrating tools and data coming from different life science fields, such as genomics or proteomics data, or spatial transcriptomics. In addition to the integration of component from different communities, one can face the challenge to run again a previously created workflow and encounter versioning problems, with time and evolution of software packages and *component* versions. Specific configuration issues also make tedious the portability for the execution of a workflow from an environment to another, such as moving between HPCs or cloud computing platforms. While the use of virtual machines accessible from a web browser to emulate a personal desktop experience may be seductive, the bioimage analysis community should not isolate itself from other communities, and in particular not from bioinformatics community. Several bioinformatics communities have already started to tackle these issues through the use of scientific workflow management systems (SWMS) ^27^^,^^28^ and standardized software packaging practices. ^29^ These SWMS have also the advantage to tackle standardized workflow description, machine-readable as well as human readable, for FAIR principles. In comparison, the usual documentation, provided when documenting a workflow in bio image analysis current practices, is usually addressed to humans (which is already laudable and not yet common practice).

One of the key elements to enable reproducibility and portability is containerization and software packaging that facilitates the reliable creation of containers. Containerization consists in embedding a piece of software, and all its dependencies and specific configuration in one file called a container image, so that the software can run consistently across different computing environments. Table 3 shows examples of workflow management systems with usage in bioimage analysis. This containerization can be performed at the level of each individual workflow component (such as in Galaxy ^30^^,^^31^), or for complete workflows (such as in BIAFLOWS ^21^ or coming to grand-challenges). Biocontainers ^32^ is proposing a standard and recipes for these containerizations, as well as a marketplace for the containers, today mostly for –omics data processing.

**Table 3.** Some example of scientific workflow management systems.

Name (SWMS)	Example of use in bioimage analysis	Reference or link
Galaxy	^2^	^31^
NextFlow	^37^^,^^38^	^39^
SnakeMake	^40^	^41^
BIAFLOWS	https://biaflows.neubias.org/#/projects (click Try online)	^21^
BioImageIT	https://bioimageit.github.io/bioimageit_gui/tutorial_pipeline.html	https://bioimageit.github.io/#/
Knime	^2^	^42^

As a community we need to join this effort for a better exploitation and reproducibility by other communities of the imaging data produced by our workflows. One of the particularities of workflows in bioimage analysis is the need for visual and accurate feedback at critical workflow steps. This human-in-the-loop requirement has so far prevented the community from using SWMS more widely. But this is now changing as image processing tools and visual feedback are now getting incorporated in SWMS. ^21^^,^^31^^,^^33^

## Towards findability and accessibility of image analysis workflows

At a general level in life-science and not specifically for the bioimage analysis community, coordination efforts are ongoing in the direction of the “FAIRification” of workflows, but also the ease to access HPC resources to run them. They are led by European Research Infrastructures, such as ELIXIR. ^34^ ELIXIR is an intergovernmental organization that aims to coordinate the resources offered nationally for databases, software tools and access to cloud storage and HPCs, and associated training material. BIII, the finder tool mentioned above, is now for example part of the recommended interoperability resources. EOSC-Life is an ESFRI cluster project involving the 13 biomedical research infrastructures whose goal is to create an open, digital and collaborative space for biological and medical research in the European Open Science Cloud. This includes making image data and image processing and analysis workflows compliant with the FAIR principles, while enabling interoperability with tools and data from other life science domains as mandated by the European Commission. Galaxy ^30^ has been identified and selected as an aggregator of communities, and selected by EOSC-Life as an exemplary workflow management system that promotes cross-communities interoperability in the cloud. This does not mean that the bioimage analysis community needs to restrict to this particular choice, but it means that the workflows have to be compatible with this choice and to prepare for a future where local compute resources will not anymore be used to run a workflow.

To ease this interoperability, a common description needs to be defined, in order to be able to make workflows interoperable and compatible with different infrastructure environments. The description of a workflow is different from the workflow itself: it is a human- and machine-readable description following standard syntax or vocabularies that will allow this workflow to be FAIR. ^35^ A workflow should be associated with a standardized description (such as a unique identifier for the workflow itself, their component, but also their creator) and a description of its constitutive components and their configuration. The researchers who created the workflow can be identified by their ORCIDs. The Common Workflow Language ^36^ could be used as a standard to describe workflows in an interoperable way since it has reached a sufficient level of maturation and flexibility. To further facilitate their findability by web search and indexing engines, lightweight metadata can be provided through the *Schema.org* controlled vocabularies or *Bioschemas* , a specific extension for Life Science resources.

Galaxy is one of many SWMS, a more exhaustive list curated by the Common Workflow Language organization can be found here. Table 3 focuses on SWMS used in the bioimage analysis field, and it details their specificities. These specificities tend to support the message that the effort should not be in trying to push the implementation of workflows in only one solution, but rather to allow and ease the portability of workflows in multiple frameworks and execution environments, an approach supported by initiatives CWL. We then argue that these standards are key to facilitate the workflow ecosystems and further promote open and reproducible sciences.

## Conclusion

The field of bioimage analysis, partly thanks to the NEUBIAS community, has been recently consolidated. Its community has contributed to the emergence of new tools to find, launch, compare and learn how to use and customize image analysis workflows. We believe that today the field has become mature enough to contribute to the general open science effort in life science and to enable better access to data and computational resources. This effort should help promote workflow sharing and reuse and a wider data integration and interoperability. We deeply encourage the bioimage analyst community, and by extension the associated software developer community, to sustain this effort and to rely on these tools. In particular, we encourage bioimage analysts to describe their workflows thoroughly by following the CWL standard, index them in BIII, and share them in SWMS such as BIAFLOWS compatible with Galaxy.

## Data availability

No data are associated with this article.
