# Supplementary material for: Bioimage analysis workflows: community resources to navigate through a complex ecosystem
Source: F1000Res. 2021 Apr 26;10:320. [Version 1] doi: 10.12688/f1000research.52569.1 (PMC8182692; doi:10.12688/f1000research.52569.1)
Supplement: Peer review history

# Peer review: Reviewer response for version 1

**DOI:** 10.5256/f1000research.55867.r84664

**Published:** 2021-06-04

**Authors:** Assaf Zaritsky

**Affiliations:** 1 Department of Software and Information Systems Engineering, Ben-Gurion University of the Negev, Beer Sheva, Israel

In this Opinion Article Paul-Gilloteaux and representatives of the Network of European BioImage AnalystS (NEUBIAS) present their views on the current challenges and solution in using bioimage analysis workflows – modular image analysis pipelines that are used to process bioimaging data. Specifically, the authors survey current approaches for finding the most appropriate workflows for a given task, evaluating and comparing workflows, reproducing results, sharing code and executing workflows remotely. The opinion is dealing with a timely and important topic, and is mostly well written and easy to follow and an enjoyable read.

 The authors present several alternative solutions for each challenge, highlighting the contribution made by NEUBIAS. This is totally legitimate since this is an opinion piece written from NEUBIAS perspective. However, I do think this point should be emphasized by including the Network of European BioImage AnalystS (NEUBIAS) in the title, by providing a brief background description about this community and by providing explicit information regarding NEUBIAS members contribution (e.g., mentioning that BIAFLOWS was developed by a NEUBIAS member).

 In my opinion, the ideas presented in the third section (“Towards findability and accessibility of image analysis workflows”) could be integrated in the previous sections. This will improve the flow of the text without losing any content. I do not see any conceptual advantage of having a separate section as in the current form.

 Perhaps the authors would consider including some of the recent platforms that make machine/deep learning applications accessible to users such as ImJoy and ZeroCostDL4Mic? It is perhaps worth mentioning the uniqueness of machine learning-based components where training is dependent on large amounts of data, but the resulting model can be lightly disseminated (however re-training with new data is adding another layer of complexity related to parameter settings in traditional workflows). 

 Another related idea that NEUBIAS is heavily involved in is training (for components development, workflow reconstruction, usage and disseminations). Perhaps the authors would be interested in including some ideas in their opinion? I recently discussed related topics ^1^.

**More specific comments, opinions and suggestions:**

 The Abstract and the beginning of the Introduction are identical. I recommend making them separate entities, where the Abstract summarizes the main ideas, while the Introductions provides more extended background and context for the rest of this piece. In the Abstract I suggest to start with the context, move to presenting NEUBIAS and workflows (do not forget to briefly explain what is a workflow) and finish with the content of this paper (I would remove the sentence on F1000 gateway, why is it relevant in the Abstract or at all?).

 Page #3 (Introduction): Since component is the building block of a workflow, I recommend defining component before workflow.

 Page #3: “We believe that while this can be a good starting point it may not be sufficient”, can you briefly mention why this is not sufficient?

 Page #4: “the natural question is then if it is the best solution for the particular task one wants to solve”. I think that most users will not find this a “natural” question, rather, their goal is to find a “good-enough” workflow to answer the question they are interested in.

 Page #5 (“Toward reproducibility”): perhaps it is worth mentioning and citing the recent paper on integrating ImageJ and CellProfiler ^2^ and/or a recent opinion reflecting on some of the aspects discussed in this section ^3^?

 Minor formatting issue: the reference comes after the period (“.”) or comma (“,”) in several locations (e.g., refs #5-7, #10, #16, #18, #24,#26).

Is the topic of the opinion article discussed accurately in the context of the current literature?

Yes

Are arguments sufficiently supported by evidence from the published literature?

Yes

Are all factual statements correct and adequately supported by citations?

Yes

Are the conclusions drawn balanced and justified on the basis of the presented arguments?

Yes

Reviewer Expertise:

Computational cell dynamics, data science in cell imaging, bioimage analysis

I confirm that I have read this submission and believe that I have an appropriate level of expertise to confirm that it is of an acceptable scientific standard.

## References

[1] : Data science in cell imaging. *J Cell Sci* .2021;134(7) : 10.1242/jcs.254292 10.1242/jcs.254292 33795377PMC8034880

[2] : ImageJ and CellProfiler: Complements in Open-Source Bioimage Analysis. *Curr Protoc* .2021;1(5) : 10.1002/cpz1.89 e89 10.1002/cpz1.89 34038030PMC8450896

[3] : Developing open-source software for bioimage analysis: opportunities and challenges. *F1000Research* .2021;10: 10.12688/f1000research.52531.1 10.12688/f1000research.52531.1 PMC822641634249339

# Peer review: Reviewer response for version 1

**DOI:** 10.5256/f1000research.55867.r83885

**Published:** 2021-05-20

**Authors:** Katy Wolstencroft

**Affiliations:** 1 Leiden Insitute of Advanced Computer Science, Leiden University, Leiden, The Netherlands

This article describes current progress in the area of creating and sharing scientific workflows for bioimage analysis. The article describes community activities that allow the collection and comparison of workflows. It also discusses the necessity for describing workflows and their components in standardised ways, using common vocabularies.

 The article serves as a gateway to bioimaging analysis workflow resources. As such, it is a good introduction and provides a clear overview. However, the reality of creating bioimaging analysis workflows, and particularly of reusing them, has some large bottlenecks. It would be beneficial to discuss, even if only briefly, what the bottlenecks are and describe current major challenges. The authors already mention interoperability and provenance as large challenges, but the connection to the data is another large challenge. Are datasets described in common formats? Are there existing ways to work with data generated with different proprietary formats? For this point, I miss a reference to the work of the Open Microscopy Environment community (e.g. ^1^).

  Are there common representations for bioimaging data? How practical is it to move such large datasets around for the purposes of analysis? For widespread use of bioimaging workflows, advances in data management and data standards must also contribute.

 Describing and sharing analysis workflows is a great step forward for the bioimage analysis community, and this paper shows that this field is maturing and gaining good traction.

Is the topic of the opinion article discussed accurately in the context of the current literature?

Partly

Are arguments sufficiently supported by evidence from the published literature?

Yes

Are all factual statements correct and adequately supported by citations?

Yes

Are the conclusions drawn balanced and justified on the basis of the presented arguments?

Yes

Reviewer Expertise:

Scientific workflows, FAIR data, semantic data integration.

I confirm that I have read this submission and believe that I have an appropriate level of expertise to confirm that it is of an acceptable scientific standard.

## References

[1] : Metadata matters: access to image data in the real world. *J Cell Biol* .2010;189(5) : 10.1083/jcb.201004104 777-82 10.1083/jcb.201004104 20513764PMC2878938
